# The semiotics of the message and the messenger: How nonverbal communication affects fairness perception

**DOI:** 10.3758/s13415-019-00738-8

**Published:** 2019-07-09

**Authors:** Michiel Spapé, Ville Harjunen, Imtiaj Ahmed, Giulio Jacucci, Niklas Ravaja

**Affiliations:** 1 Department of Psychology, Liverpool Hope University, Liverpool, UK; 2 Helsinki Institute for Information Technology HIIT, Department of Computer Science, University of Helsinki, Helsinki, Finland; 3 Department of Psychology and Logopedics, University of Helsinki, Helsinki, Finland; 4 Department of Information and Service Management, Aalto University, Helsinki, Finland; 5 Department of Social Research, University of Helsinki, Helsinki, Finland; 6 Department of Computer Science, Aalto University, Espoo, Finland

**Keywords:** EEG, ERP, MFN, Economic decision-making, Nonverbal communication, Emotional expressions, Touch, Neurosemiotics

## Abstract

Nonverbal communication determines much of how we perceive explicit, verbal messages. Facial expressions and social touch, for example, influence affinity and conformity. To understand the interaction between nonverbal and verbal information, we studied how the psychophysiological time-course of semiotics—the decoding of the meaning of a message—is altered by interpersonal touch and facial expressions. A virtual-reality-based economic decision-making game, ultimatum, was used to investigate how participants perceived, and responded to, financial offers of variable levels of fairness. In line with previous studies, unfair offers evoked medial frontal negativity (MFN) within the N2 time window, which has been interpreted as reflecting an emotional reaction to violated social norms. Contrary to this emotional interpretation of the MFN, however, nonverbal signals did not modulate the MFN component, only affecting fairness perception during the P3 component. This suggests that the nonverbal context affects the late, but not the early, stage of fairness perception. We discuss the implications of the semiotics of the message and the messenger as a process by which parallel information sources of “who says what” are integrated in reverse order: of the message, then the messenger.

**Electronic supplementary material:**

The online version of this article (10.3758/s13415-019-00738-8) contains supplementary material, which is available to authorized users.

A common myth is that “in every social encounter, nearly two-thirds of the interaction meaning is derived through nonverbal messages, pp. 115” (e.g., Ting-Toomey, 1999). This is an extreme overinterpretation of a classic series of studies by Mehrabian (1971). Mehrabian and Ferris (1967), for instance, showed that an attitude towards a stranger saying “maybe” was approximately 1.5 times more affected by seeing the stranger’s facial expression than by their tone of voice. Another key study showed that participants placed more emphasis on the tone of voice than on verbal content whilst rating the attitude of one stranger speaking to another (Mehrabian & Wiener, 1967). These findings led to the famous equation that communicating attitude (feeling or liking) equals 55% body language + 38% tone of voice + 7% language (Mehrabian, 1971, pp. 43–44). However, such definite numbers of course belie the extreme breadth and complexity of communication: How we evaluate a message can well be defined by its contents (e.g., a demand for tax vs a profession of love) more than by the body language it is accompanied by. And yet, it is clear that nonverbal context does influence appraisal: Emotional responses to television messages are affected by presentation attributes, such as colour, motion, and screen size (Detenber, Simons, & Reiss, 2000; Ravaja, 2004; Reeves, Lang, Kim, & Tatar, 1999).

Determining what a message signifies—the semiotics—thus depends on a variety of modalities and contextual factors and must rely on a network of different neural structures that drive an interplay of cognitive functions. For example, language can dramatically affect emotion perception (Barrett, Lindquist, & Gendron, 2007), and emotional context determines which brain areas are activated while understanding short sentences as either literal or ironic (Eviatar & Just, 2006). A similar finding that is of particular present relevance is that receiving a message of an unfair proposal in an economic decision-making game elicits activity in areas related to emotion as well as cognition (Sanfey, Rilling, Aronson, Nystrom, & Cohen, 2003). Likewise, the facial appearance of the bringers of offers, and the mood of those who received them, was found to determine how such messages were processed (Ma, Qian, Hu, & Wang, 2017; Riepl, Mussel, Osinsky, & Hewig, 2016).

Yet, while it is widely accepted that emotional processes and nonverbal behaviour contribute to behaviour, surprisingly little is known about the cognitive neurodynamics that determine the interplay between a message and its nonverbal context, as provided by its messenger**.** If nonverbal context affects how a message is evaluated, then neural processes associated with the evaluation should be critically determined by behaviour of the messenger. To some extent, this has been studied using priming paradigms in which both prime (the context; e.g., a smiling messenger) and probe (e.g., a positive stimulus) have affective content. Event-related potentials studies show, however, that the emotional context sometimes does (Ravaja, Harjunen, Ahmed, Jacucci, & Spapé, 2017), and sometimes does not, modulate the emotional processing of the message (Schupp, Schmälzle, Flaisch, Weike, & Hamm, 2013). Of course, in real social situations, as opposed to priming paradigm, the nonverbal context and message are both related to a single source—the *messenger*. Because the messenger displays a certain emotional state (e.g., anger), we may expect a negative message, resulting in a top-down, biased perception of the actual communication. Conversely, it is equally possible that the context is only processed after the message itself is evaluated—for example, due to the operation of feature integration mechanism (Kahneman, Treisman, & Gibbs, 1992; Treisman, 1996).

## The message: The neurodynamics of message evaluation in the ultimatum game

To investigate the neurodynamics of how a messenger’s nonverbal behaviour affects the semiotics of a message, we used the classic behavioural economics game, ultimatum (Güth, Schmittberger, & Schwarze, 1982). In the ultimatum game, as also used by the aforementioned Sanfey et al. (2003), participants respond to offers from a hidden *proposer*, who decides how a given amount of money is divided between the two. If the participant agrees, each gets their pay-out, while declining an offer results in an outcome of nothing for either party. While economically costly, rejection of unfair offers is a common finding in the ultimatum game, which implies a critical role of irrational motives (von Neumann & Morgenstern, 1944). Due to this, the ultimatum game provides a powerful standard to operationalize social, cognitive, and affective contributions to message evaluation by studying their influences on offer rejection. Studies of event-related potentials (ERPs) add to this by revealing the temporal dynamics of emotional evaluation of offers (Boksem & De Cremer, 2010).

Boksem and De Cremer (2010), following Polezzi et al. (2008), used this economic decision-making game and showed that perceiving an unfair offer results in medial frontal negativity (MFN), which they related to a matching process between a social norm of fairness, and the present reality of unfairness. The MFN is, beyond the literature on the ultimatum game, related to a broad class of ERP components that describe a type of error, such as with incorrect responses indexed by the error-related negativity (ERN; Gehring, Goss, Coles, Meyer, & Donchin, 1993), incongruence between primed and task-relevant actions resulting in the N2b (Nieuwenhuis, Yeung, van den Wildenberg, & Ridderinkhof, 2003), and feedback indicating a loss or failure indexed by the feedback-related negativity (FRN, Gehring & Willoughby, 2002).^1^ These components are similar in functional, topographical and temporal characteristics in the sense that all involve performance monitoring functions, a similar fronto-centrally negative topography, and a common latency of at least 200 ms post stimulus (Folstein & Van Petten, 2008). This has led to the suggestion that the same, anterior cingulate located neural generator is activated whenever performance seems “worse than expected”, irrespective of paradigm, giving rise to both the ERN and the FRN/MFN (Holroyd & Coles, 2002; but see Gehring & Willoughby, 2004).

Aside from the MFN, the message context may affect emotional evaluation during other stages of stimulus processing as well. Preceding the MFN by about 100 ms, the N1 is the first clear negative visual evoked potential and is characterised by a predominantly frontal topography and an onset at ca. 100 ms (Harter & Previc, 1978; Spitz, Emerson, & Pedley, 1986). Previous studies have shown it is enhanced in response to emotionally relevant stimuli, which may be due to an early attentional mechanism that prioritises evolutionary salient pleasant and unpleasant arousing stimuli (Schupp, Junghöfer, Weike, & Hamm, 2003), such as dangerous animals and fearful facial expressions (Zhang, Luo, & Luo, 2013). While there are few studies explicitly measuring the effect of nonverbal contexts on the N1, Schirmer et al. (2010) found a particular effect of interpersonal touch on this early component, showing that a simple touch enhances the N1 to emotion-evoking pictures, potentially arguing in favour of a top-down effect of context on salience-related processing.

Peaking after the MFN, the P3 is a complex, late potential that is characterised by three components: the P3a, the P3b, and the late positive potential (LPP). The P3a commonly has a more frontal topography, a somewhat earlier latency (at ca. 300 ms), and is sometimes referred to as the “novelty”-related P3 (Friedman, Cycowicz, & Gaeta, 2001). The P3b (or “classic P300”), in contrast, has a more parietal topography, and is commonly identified, but enhanced if a stimulus is unexpected, meaningful, or task-relevant (Squires, Squires, & Hillyard, 1975; Sutton, Braren, Zubin, & John, 1965; Sutton, Tueting, Zubin, & John, 1967). More variegated emotional differentiation can often be observed in this interval—for instance, with fearful and happy facial emotional expressions distinctly affecting the P3 potential in emotion classification tasks (Luo, Feng, He, Wang, & Luo, 2010; Spapé, Harjunen, & Ravaja, 2017).

Finally, the LPP is not always clearly distinguishable from the P3a or P3b, and has been identified at frontal as well as parietal sites, and a latency following the initial P300 (Krolak-Salmon, Fischer, Vighetto, & Mauguière, 2001; Schupp et al., 2004). The LPP has been found to be sensitive to faces requiring more elaborate processing, and its measurement allows discrimination between different emotional expressions such as happiness, anger, fear, surprise, and disgust (Krolak-Salmon et al., 2001; Schupp et al., 2004; Spapé et al., 2017). Such more nuanced emotional appraisal might define a stimulus in an approach–avoidance type of dimension, which has been related to the LPP (Bamford et al., 2015; Gable & Harmon-Jones, 2010). Likewise, in the context of the ultimatum game, it was found that the LPP was sensitive to a more socially qualified sense of unfairness: Unfairness amplified the LPP only if others were (supposedly) treated even worse than the participant (Wu, Zhou, van Dijk, Leliveld, & Zhou, 2011).

## The messenger: Two channels of nonverbal communication

To study how nonverbal behaviour affects semiotics, we investigate *whether* and *when* two common nonverbal channels—emotional expressions and interpersonal touch—modulate the difference between processing of fair and unfair offers in the ultimatum game, also referred to as fairness perception (Moser, Gaertig, & Ruz, 2014). Accumulating evidence suggests that seeing another person smiling or frowning potentiates a perceiver’s attention and early sensory-perceptual processes (Hinojosa, Mercado, & Carretié, 2015; Luo et al., 2010). As a result, emotional expressions can have strong effects on cognition and, ultimately, decision-making. Proposals accompanied by smiles are more likely to result in signed petitions (Vrugt, 2007), help in picking up dropped items (Guéguen & De Gail, 2003), and accepted offers in the ultimatum game (Mussel, Göritz, & Hewig, 2013).

Another common nonverbal channel of communication that has received much interest in affective neuroscience and decision-making is touch. Touch may be a particularly influential social cue due to its necessarily intimate nature and sensitivity to other contextual factors (Gazzola et al., 2012; Ravaja, Harjunen, Ahmed, Jacucci, & Spapé, 2017). For instance, even a causal touch from a stranger has been found to increase appreciation, prosociality, and compliance in the receiver (Fisher, Rytting, & Heslin, 1976; Goldman & Fordyce, 1983; Guéguen & Fischer-Lokou, 2003). Crusco and Wetzel (1984), who first demonstrated this *Midas Touch* effect, found that waiters touching their customers during the service got larger tips than those who did not touch. The effect has since been replicated in various field and laboratory studies (Gallace & Spence, 2010). It is thus clear that both facial expressions and touch do affect receivers’ decisions, even if the underlying neural mechanisms and the temporal dynamics of the semiotic modulation remain obscure.

## The message and the messenger: Present study

In order to investigate how the nonverbal behaviour of a messenger affects the evaluation of their message, it is essential to present both aspects as part of the same embodied source. As we argued elsewhere (Ravaja et al., 2017), this is not usually feasible in a classic laboratory setup: Presenting a touch followed by an offer is not the same experience as showing a person “touching you” and *the same person then* making an offer. To enable the latter scenario, we used virtual reality presenting both the emotional expression and touch originating from the same artificial agent.

The principal aim of the study was to find out whether and when the nonverbal channels would modulate offer evaluation. If nonverbal channels do not affect offer perception at all, then they should not interact with the message content (the type of offer) in terms of their effect on ERP components. In this case, the nonverbal context could still be observable as main effects of message context—for example, touch might amplify salience detection, resulting in a generally increased N1 (Schirmer et al., 2010). Of more interest, however, would be evidence of biased offer perception observed as interactions between nonverbal context and message content. These should not be observed on N1, as message content is theoretically not yet decoded at this stage. However, an immediate interaction between fairness and context found on the MFN could indicate a top-down influence. For example, if a smile or touch promotes a prediction of a positive message, then unfair offers should be perceived as “worse than expected” (Holroyd & Coles, 2002), consequently incurring a stronger MFN to unfair relative to fair offers. On the other hand, if the nonverbal context affects later, bottom-up appraisal processes, the interpretation of the unfair offer could conflict with its smiling context, resulting in enhanced requirements for context updating (Donchin & Coles, 1988), and therefore affecting the P3 or LPP components.

## Method

### Participants

Thirty-one male and 35 female participants were recruited for the experiment. They were all students from the University of Helsinki and Aalto University, with a mean age of 24.37 (*SD* = 3.45, range: 19–46) years and with no history of neurological or psychiatric disorders. The participants were briefed about the purpose of the experiment to the extent that the study concerned social decision-making in virtual reality. The participants were also told that the agents’ behaviour was guided by an algorithm approximating human behaviour in similar situation. Before asking to sign the informed consent and commencing the experiment, participants were informed about their right to withdraw from the study at any time without fear of negative consequences. The study was conducted in accordance with the guidelines set out in the Declaration of Helsinki and was approved by the Aalto Ethic Committee. The amount of money they earned as part of the ultimatum game (*M* = 39.91, *SD* = 3.45 euros) was paid to the participant as a compensation for their participation; or, in case the cumulative earnings were below a minimum compensation of 35, the amount of 35 euros was paid.

### Stimuli and apparatus

Tactile stimuli were presented using a haptic glove, which used a motor to tighten elastic fabric over the hand. Technical details of the custom glove are provided by Ahmed et al. (2016), who found that this type of tactile feedback was judged as more natural and better suited to computer-mediated tactile communication than traditional vibrotactile actuators.

Visual stimuli were presented via a head-mounted display (HMD, Oculus Rift DK2), which used positional tracking, stereoscopic displays (1,920 × 1,200 pixels per eye; 75-Hz refresh rate; 100° nominal field of view) and parallax cues to provide an immersive visual experience. A similar tactile-augmented setup was used by Ravaja et al. (2017), who likewise portrayed virtual agents to present emotional expressions. However, instead of the one agent used by Ravaja et al. (2017), we used eight different agents to improve the impression that each ultimatum game scenario was distinct from the previous one (similar to de Melo, Gratch, Carnevale, & Read, 2012). The 3-D models of the agents were manually morphed from Genesis 2 male and female characters of Daz Studio (Daz Productions Inc., Salt Lake City, UT), and modelled after real-life male and female people from four ethnic backgrounds (European, African, Southeast Asian, and Central Asian). Dynamic emotional expressions were designed using Unity 3D 4.5.4 software (Unity Technologies, San Francisco, CA), manipulating the facial action units involved in prototypical expressions of happiness and anger (Ekman & Friesen, 1971).

An Intel-based desktop PC was used to run custom software, designed using Unity 3D software, to control stimulus presentation, recording of reactions, and communication with the EEG amplifier. A photo sensor and an accelerometer were used to additionally record the onset of visual and tactile stimuli in order to improve the synchronization and validate timing accuracy. Timing, topography, and signal-to-noise ratio of the virtual reality setup were recently tested and validated in a traditional oddball experiment (Harjunen, Ahmed, Jacucci, Ravaja, & Spapé, 2017).

### Procedure

After receiving instructions, signing of informed consent, and setting up of the EEG equipment, participants received assistance in putting on the HMD and fitting on the tactile glove. The experiment comprised one training block of 18 trials, and eight experimental blocks of 72 trials. Upon completing a block, participants were asked to take a short self-timed break, during which they also received feedback on how much money they had accumulated thus far. During training, participants received additional instructions on the need to avoid movements and on the need to respond soon after but not during presentation of the offers. They received full debriefing after the final block, which was 83.5 (*SD* = 10.6) minutes after the start of the experiment on average.

**Fig. 1:**
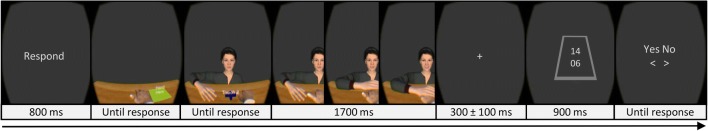
Schematic presentation of a trial sequence. (Colour figure online)

**Fig. 2:**
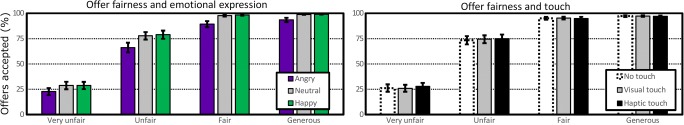
Behavioural effects. Left: Offer acceptance as a function of fairness of offer and emotional expression. Right: Offer acceptance as a function of fairness and interpersonal touch. Error bars show standard error of means. (Colour figure online)

Figures 1 and 2 summarizes the presentation and timing of events during experimental trials. Trials started with the word “Respond”, cuing the role of the participant as a responder, or “Propose”, as a proposer. The latter were included in view that previous studies (Boksem & De Cremer, 2010; Spapé, Hoggan, Jacucci, & Ravaja, 2015) used similar schemes to enhance the realism of responder trials; they were, however, not further considered for analysis. Following a duration of 4,000 ms, for the trials with a new role, or 800 ms for every subsequent one, the interaction scenario was presented: a table showing a green area to the right, and the participant’s virtual hand. Moving the hand over the green area was used as a trigger to show the virtual agent, as a well as a blue manual fixation cross hair. Moving their hand over this position started 0–200 ms of random delay, followed by a 1,000-ms animation in which the emotional expression (anger, neutral, happiness) and physical interaction (none, visual touch, tactile touch) was dynamically displayed. In the visual and tactile touch conditions, the virtual hand moved towards the participant’s, reaching it at 1,000 ms. In the tactile touch condition, this additionally started 500 ms of tactile feedback using the glove. In all three conditions, the animation was otherwise static for 500–700 ms. The interaction scenario was then replaced by a fixation cross hair with a duration of 100–300 ms (randomized), before the proposal from the agent was shown. The proposal was shown as two numbers—the upper one representing the amount for the agent and the lower the amount for the subject—with a frame around the two numbers to indicate that the upper number referred to the person on the other end of the table. The proposal was always shown for 900 ms, followed by a response cue. Pressing the left button on the keypad (below the participant’s left hand) would accept the response, while the right button would reject it. The next trial was presented after a blank intertrial interval of 500 ms.

### Design

The experiment consisted of eight blocks of 72 trials. In each block, the three types of touch (no touch, visual touch, tactile touch), three types of emotional expression (angry, neutral, happy), and four types of offers (very unfair: 2|18, 3|17, 4|16, 5|15, 6|14; unfair: 7|13, 8|12; fair: 10|10; generous: 11|9, 12|8, 13|7, 14|6, 15|5) were randomly mixed and repeated twice. The pool of offer sizes was based on Boksem and De Cremer (2010), who used a skewed distribution such that the probability of more equal offers was higher than less equal ones. In the present study, 6.7% were 2|18 offers, 13.3% were 3|17, 20.0% were 4|16, 26.7% were 5|15, and 33.3% were 6|14; of unfair offers, 46.2% were 7|13 and 53.8% were 8|12. Boksem and De Cremer (2010) did not use generous offers, so each specific offer was provided 20% of times. In each block, the first, second, third, or fourth 18 trials were randomly selected to appear as proposer trials. Each block was further subdivided into four series of 18 trials each, with one (random) series comprising proposer trials and the others responder trials. Statistical analyses were run over the 432 responder trials, with ERPs calculated over a maximum of 36 trials, for two repeated measures ANOVAs: one with offer size (four levels) and emotional expression (three levels), and one with offer size, and touch (three levels).

### EEG recording and preprocessing

A QuickAmp (BrainProducts GmbH, Gilching, Germany) amplifier was used to record EEG at 1000 Hz with a hardware band-pass filter from 0.01 to 500 Hz from equidistantly placed Ag/AgCl scalp electrodes, positioned using EasyCap elastic hats (EasyCap GmbH, Herrschin, Germany). EEG was recorded from sites overlying Fp1, Fp2, F7, F3, Fz, F4, F8, FT9, FC5, FC1, FC2, FC6, T7, C3, Cz, C4, T8, TP9, CP5, CP1, CP2, CP6, TP10, P7, P3, Pz, P4, P8, O1, Oz, and O2 of the 10–10 system (Chatrian, Lettich, & Nelson, 1985). Horizontal and vertical electro-oculographic (EOG) activity was recorded using two pairs of electrodes, respectively placed 1 cm laterally to both eyes, and superior and inferior to the right eye.

EEG preprocessing included a band-pass filter between 0.2 and 80 Hz with a notch filter at 50 Hz. Artefact correction used independent component analysis (ICA) using the logistic infomax algorithm as implemented in EEGLAB (Delorme & Makeig, 2004), with extended parameters (learning rate = .001, learning rate lowered by 2% if angle Δ < 60^o^, learning ending after 512 steps or weight change < 1E07. For full code, see EEGLAB runica algorithm Version 25 January 2002, https://github.com/sccn/eeglab). The ICA was run on epochs of 7 s, time-locked to the offer onset, with 3.5 s of baseline activity. Following this, we visually inspected the components for the presence of abnormal frequency spectra, topographies suggesting ocular dipoles, and the absence of event-related activity. The source-level EEG was then reconstructed by applying the artefact-free weights to the unfiltered, continuous data.

Following the artefact correction, we followed common steps in the MFN/ultimatum game literature (e.g., Boksem & De Cremer, 2010; Van der Veen & Sahibdin, 2011; Wu, Zhou, van Dijk, Leliveld, & Zhou, 2011): applying a linked mastoid reference and a 40-Hz low-pass filter before segmenting the data into 1-s epochs, time-locked to the onset of the offer and including 200 ms of baseline activity. To further remove the effects of artefacts, we applied an individually tailored threshold-based artefact rejection procedure that used a stair-climbing procedure, which removed on average 4.6 (*SD* = 4.8) % of epochs by applying a maximum threshold between 27 μV and 68 μV and a maximum max-min difference between 27 μV and 90 μV. Following removal procedures based on behaviour (see below) and of participants who had in any design cell fewer than 25 epochs, we used an average number of 34.5 (*SD* = 1.2) epochs per design cell to calculate individual ERPs.

### ERP time-window definition and analysis

**Fig. 3:**
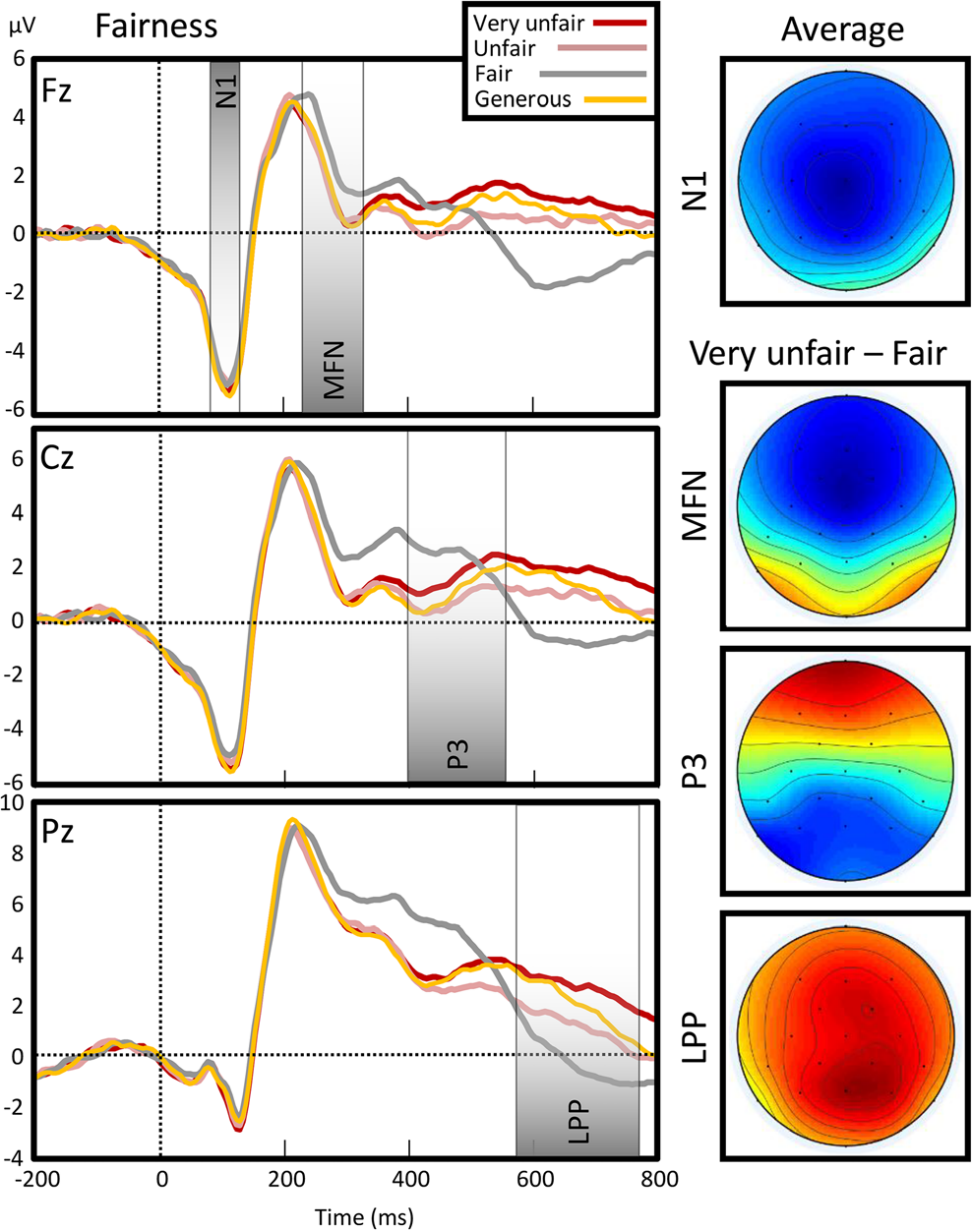
Event-related potential to the offer as a function of fairness. Topographies show distribution of voltage for the general N1 potential (averaged across conditions) and for the fairness effect in the medial frontal negativity (MFN), P3 and late positive potential (LPP) intervals. The fairness effect was calculated as the difference between very unfair (red) and fair (grey) offers. (Colour figure online)

Consistent with the literature on the FRN/MFN (Holroyd & Coles, 2002; Van der Veen & Sahibdin, 2011; Yeung, Botvinick, & Cohen, 2004), we focussed the analysis on midline frontal electrodes Fz and Cz, and included the Pz for additional investigation of the P3. The N1 was defined as the first clearly visible, negative peak with a frontal topography, defined over the average across conditions as the first local minimum. This was observed at 105 ms (−4.76 μV), corresponding to previous studies (Holmes, Vuilleumier, & Eimer, 2003), and we defined a 50-ms window centred on this latency (80–130 ms) as the measurement of N1. We used a data-driven approach, partially inspired by Boksem and De Cremer (2010), to define the MFN. As the analysis concerned the modulation (rather than the main effect) of the MFN and later potentials, we used a windowed repeated-measures ANOVA ,with fairness (very unfair, unfair, fair, and generous) as the factor and mean voltage of 10-ms bins as the dependent variable. This showed an early effect from 230 ms to 350 ms over Fz (peaking at 250 ms), *F*(2, 56) = 11.84, *p* < .001. As will be seen from Fig. 3, the fair condition contributed much stronger to the differences than any other condition. Thus, after removing the fair condition from the analysis, the earliest effects of fair offers shifted to 400–600 in Fz, while later effects were observed in Cz and Pz (both from 530 to 780). Accordingly, we used three windows that defined the classic MFN (230 to 350)—in which there was any effect of fairness—and later windows that had effects of offer size. As the earlier part of this effect of offer size showed a more frontal and the latter a more parietal topography, we defined these as, respectively, P3 (400–580) and LPP (580–780). The analyses for each window were based on 36 amplitude averages, as with three emotional expressions (happy, neutral, angry) *or* three types of touch (no touch, visual touch, tactile touch), four offer sizes (very unfair, unfair, fair, and generous), and three electrodes (Fz, Cz, Pz).

### Statistical analysis

Three sets of analyses were conducted. We first investigated the behavioural effects (see the section titled Effects of Emotional Expression and Touch on Behaviour) within a single repeated-measures ANOVA, with emotional expression, touch, and offer size as factors, and acceptance as the dependent variable. The focus of the present study constituted the analysis of the ERPs. For this, we ran two repeated-measures ANOVAs: one to investigate the effect of emotional expression on offer perception (see the section titled Effects of Emotional Expression and Fairness on the ERP), and the second to investigate the effect of touch on offer perception (see the section titled Effects of Touch on Offer Perception). This approach allowed us to ascertain sufficient number of epochs per design cell for ERP analysis, and to reduce the likelihood of Type I error reporting.^2^ As we made no specific hypothesis that the emotional expression would influence the effect that touch has on offer evaluation, and as such the hypothesis was not supported by the behavioural evidence reported further, we decided to reduce the complexity towards two four-way repeated-measures ANOVAs, testing against an adjusted significance level of *p* < .0125 (see the section titled Effects of Emotional Expression and Fairness on the ERP). First, an ANOVA was conducted, with emotional expression (anger, neutral, happiness), fairness (very unfair, unfair, fair, generous), electrode (Fz, Cz, Pz), and time (N1, MFN, P3, LPP) as factors. To analyse the time-course of effects, each time window was further investigated with three-way interactions. A second, similar ANOVA was run, but with touch (none, visual, visuo-tactile), offer size, electrode, and time as factors. Regarding *fairness,* it will become clear over the course of the analysis that *fair* offers show a pronounced contrast with all other offer types. To dissociate the singular effect of *fairness* from the more variegated difference between various offers, we follow up any significant effect of *fairness* with the same analysis but exclude the *fair* level. We refer to this follow-up analysis as investigating the factor of *offer size* (very unfair, unfair, generous). Main effects of electrode are not reported, as are redundant effects in common between conditions (e.g., effects of offer size). Greenhouse–Geisser correction is applied when necessary. Nonsignificant, theoretically interesting findings are reported along with observed power estimates.

## Results

An initial analysis of the behavioural and EEG data was used to determine whether participants should be excluded from the data set. Three participants accepted more than 95% of offers in the two unfair conditions. Another three participants were removed for accepting substantially (10%) fewer generous than fair offers. Finally, three participants were excluded for having fewer than 25 epochs per design cell left following artefact rejection. The final sample consisted of 57 participants (33 females, 24 males), age *M* = 24.37, *SD* = 4.98 years.

### Effects of emotional expression and touch on behaviour

A repeated-measures ANOVA with *fairness* (very unfair, unfair, fair, and general), *touch* (none, visual touch, tactile touch), and *emotional expression* on *acceptance* (%) showed significant effects of *fairness*, *F*(2.05, 114.88) = 205.06, *MSE* = 3920.53, *p* < .001, ${\eta}_p^2$ = .79, and *emotional expression*, *F*(1.12, 62.76) = 15.14, *MSE* = 1778.57, *p* < .001, ${\eta}_p^2$ = .21, but not *touch*, *F*(1.81, 101.24) = 1.29, *MSE* = 75.59, *p* = .28, ${\eta}_p^2$ = .02, power = .26. Planned comparisons with incremental levels of fairness showed that unfair offers were accepted more often (74.20%) than very unfair ones (26.65%), *t*(56) = 13.34, *p* < .001, and fair offers (95.04%) more often than unfair offers. Generous offers (97.06%) were not significantly more often accepted than fair offers, *p* = .07. Contrasts of the emotional expressions versus the neutral condition showed the main effect of *emotional expression* to primarily indicate that fewer offers were accepted after angry (67.82%) than neutral (75.72%) emotional expressions, *t*(56) = 4.06, *p* < .001. Happy emotional expressions did not significantly result in more offers being accepted, p = .45.

A significant interaction of *emotional expression* and *fairness* was observed, *F*(3.24, 181.20) = 2.98, *MSE* = 341.39, *p* = .029, ${\eta}_p^2$ = .05. As shown in the left panel of Fig. 2, the effect indicated an increased difference between angry and happy as well as angry and neutral expression conditions in fair (happy–angry = 9.06%; neutral–angry = 8.54%) and unfair condition (happy–angry = 12.73%; neutral–angry = 11.66%), as compared with very unfair (happy–angry = 5.98%; neutral–angry = 5.96%) and generous condition (happy–angry = 5.63%; neutral–angry = 5.43%). The predicted interaction between *fairness* and *touch* was not observed, *p* = .40, ${\eta}_p^2$ = .02, power = .41, nor did *touch* interact with *emotional expression*, *p* = .68, ${\eta}_p^2$ = .01, power = .19, or enter a three-way interaction with both *emotional expression* and *fairness, p* = .82, ${\eta}_p^2$ = .01, power = .37.

### Effects of emotional expression and fairness on the ERP

A repeated-measures ANOVA on the average amplitude, with *time* (N1, MFN, P3, LPP), *electrode* (Fz, Cz, Pz), *emotional expression* (angry, neutral, happy), and *fairness* (very unfair, unfair, fair, generous) showed a significant effect of time, *F*(2.42, 1.31) = 103.49, *MSE* = 223.11, *p* < .001, ${\eta}_p^2$ = .65, *emotional expression*, *F*(1.88, 105.18) = 14.95, *MSE* = 1080.85, *p* < .001, ${\eta}_p^2$ = .21, but not offer size, *p* = .06. *Time* interacted with *fairness*, *F*(5.16, 288.89) = 34.37, *MSE* = 14.44, *p* < .001, ${\eta}_p^2$ = .38, and *emotional expression*, *F*(4.69, 262.53) = 6.28, *MSE* = 4.24, *p* < .001, ${\eta}_p^2$ = .10. The interaction between *emotional expression* and *fairness* was not significant, *p* = .054, while the three-way interaction between *time, emotional expression,* and *fairness* did not show a significant effect, *F*(10.97, 614.41) = 2.54, *MSE* = 4.30, *p* = .004, ${\eta}_p^2$ = .04. Therefore, the effects of *emotional expression*, *fairness,* and the interaction between the two factors, played out over different potentials, for which reason we split the above analysis into ANOVAs and separately examined the N1, MFN, P300, and LPP with alpha level at *p* < .0125 (5%/4) to adjust for Type I errors.

#### Effects of emotional expression and fairness on N1

A repeated-measures ANOVA on average amplitude over the N1 interval with *electrode* (Fz, Cz, Pz), *fairness* (very unfair, unfair, fair, generous), and *emotional expression* (angry, neutral, happy) showed that neither *fairness, p* = .024, ${\boldsymbol{\eta}}_{\boldsymbol{p}}^{\mathbf{2}}$ = .06, power = .51, nor *emotional expression*, *p* = .016, ${\boldsymbol{\eta}}_{\boldsymbol{p}}^{\mathbf{2}}$ = .07, power = .53, was significant (given α = .0125; see the section titled Effects of Emotional Expression and Fairness on the ERP).

#### Effects of emotional expression and fairness on MFN

A repeated-measures ANOVA on average amplitude over the MFN interval, with *electrode* (Fz, Cz, Pz), *fairness* (very unfair, unfair, fair, generous), and *emotional expression* (angry, neutral, happy), showed that *fairness* was significant*, F*(2.55, 142.79) = 16.93, *MSE* = 11.25, *p* < .001, ${\boldsymbol{\eta}}_{\boldsymbol{p}}^{\mathbf{2}}$ = .23, as was *emotional expression, F*(1.81, 101.26) = 8.63, *MSE* = 11.55, *p* = .001, ${\boldsymbol{\eta}}_{\boldsymbol{p}}^{\mathbf{2}}$ = .13. *Emotional expression* furthermore interacted with *electrode*, *F*(2.55, 142.58) = 5.17, *MSE* = 1.21, *p* = .003, ${\boldsymbol{\eta}}_{\boldsymbol{p}}^{\mathbf{2}}$ = .08. *Emotional expression* was found to mainly signify a difference between the two emotional conditions (angry = 3.63 μV, happy = 3.76 μV) and neutral (3.08 μV). The interaction with *electrode* showed the effect to apply more to the central (0.81 μV) and frontal (0.68 μV) electrodes than the parietal one (0.37 μV).

Of more interest was the effect of *fairness*: This showed mainly an effect of *fair* offers evoking more positivity than unfair ones provoking negativity (as is the default description). That is, while unfair and very unfair (3.18 μV and 3.22 μV) evoked negativity versus fair (4.33 μV) offers, so did generous offers (3.22 μV). This exclusive, positive effect of fairness, relative to the negative effect of generous offers, prompted the analysis referred to in 2.7 as the analysis of *offer size—*that is, following up any analysis showing a significant effect of *fairness* with one that included all offer types but for the fair one. Here, the effect of *offer size* (very unfair, fair, vs generous offers) was nonsignificant, *p* = .95, ${\eta}_p^2$ = .001, power = .02, as was the interaction between *offer size* and *electrode*, *p* = .92, ${\eta}_p^2$ = .004, power = .03. Put next to one another, the analyses of *fairness* and *offer size* thus reveal the effect of fairness is exclusively defined by *fair offer specific activity.*

#### Effects of emotional expression and fairness on P3

**Fig. 4:**
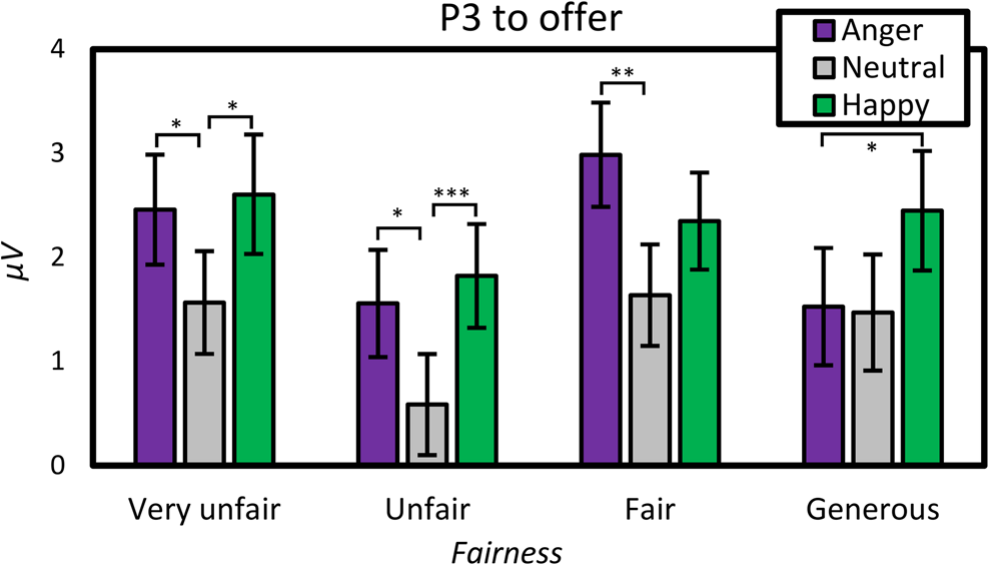
Effects of offer and emotional expression on P3. P3 calculated as the amplitude average over Fz, Cz, and Pz electrodes. Error bars show standard error of means. (Colour figure online)

**Fig. 5:**
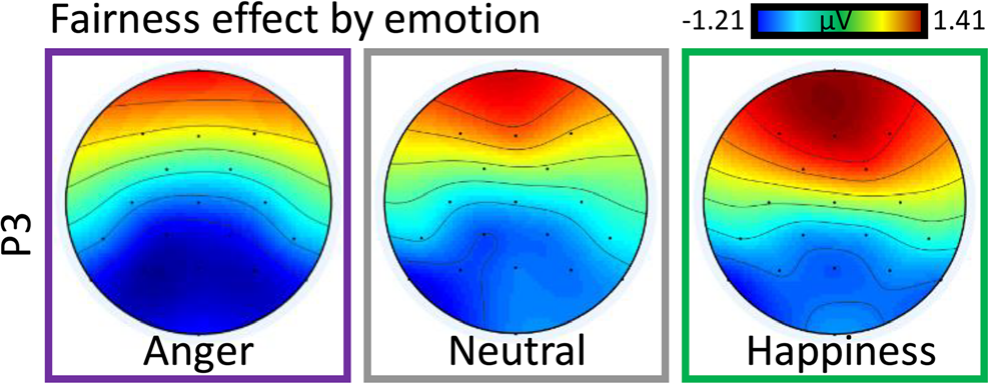
Emotional expression of the agent modulates fairness perception at the P3 window. The fairness effect was calculated as the difference between very unfair and fair conditions. (Colour figure online)

Repeated measures ANOVAs on the average amplitude of the P3 interval, with *electrode, fairness,* and *emotional expression* showed significant effects of *fairness*, *F*(2.45, 137.35) = 5.69, *MSE* = 22.69, *p* = .002, ${\boldsymbol{\eta}}_{\boldsymbol{p}}^{\mathbf{2}}$ = .09, and *emotional expression*, *F*(1.81, 101.32) = 16.49, *MSE* = 12.87, *p* < .001, ${\boldsymbol{\eta}}_{\boldsymbol{p}}^{\mathbf{2}}$ = .23. Fairness showed maximum negativity with unfair offers (1.32 μV) followed by generous (1.82 μV), very unfair (2.21 μV), and fair (2.32 μV) ones. *Emotional expression* showed similar effects to those on MFN, with neutral expressions (1.31 μV) evoking a lower amplitude than angry (2.13 μV) and happy (2.31 μV) expressions. Moreover, fairness interacted with electrode, *F*(3.42, 191.45) = 16.33, *MSE* = 3.10, *p* < .001, ${\boldsymbol{\eta}}_{\boldsymbol{p}}^{\mathbf{2}}$ = .23. Fair offers now evoked positivity over the parietal (4.32 μV in fair vs 2.67 μV in unfair conditions) and central (2.05 μV vs 0.82 μV) sites, but not over the frontal (1.45 μV in very unfair vs 0.61 μV in fair conditions) one. *Emotional expression* also interacted with fairness, *F*(5.37, 300.93) = 3.62, *MSE* = 6.63, *p* = .003, ${\boldsymbol{\eta}}_{\boldsymbol{p}}^{\mathbf{2}}$ = .06. As shown in Fig. 4, the effect showed positivity to be strongest for angry/fair (2.99 μV) and happy/very unfair (2.61 μV) offers and weakest for neutral/unfair (0.59 μV) conditions. To further illustrate this interaction between *emotional expression* and *fairness*, we calculated the fairness effect for each electrode as the difference between *very unfair* and *fair* conditions and show the scalp topography of this effect as a function of *emotional expression.* As can be seen in Fig. 3, this shows that happy emotional expression amplified the frontal part of the P3, while attenuating the parietal part (Figs. 5).

**Fig. 6:**
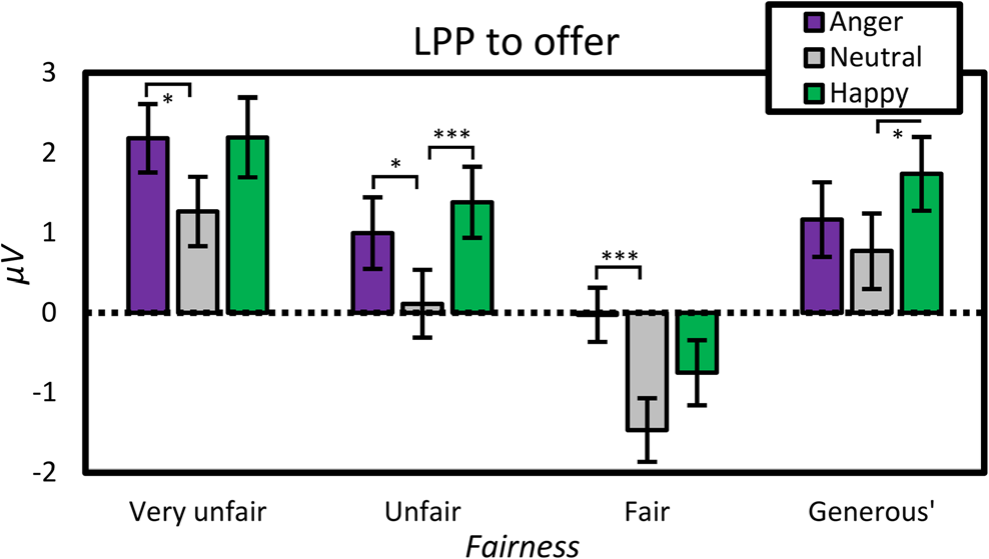
Effects of offer and emotional expression on LPP. LPP calculated as the amplitude average over Fz, Cz, and Pz electrodes. Error bars show standard error of means. (Colour figure online)

In contrast to the MFN analysis, substituting the *fairness* for the *offer size* factor did not change its significance, *F*(1.97, 110.46) = 7.74, *MSE* = 13.31, *p* = .001, ${\eta}_p^2$ = .12. However, *offer size* no longer interacted with *emotional expression*, *p* = .09.

#### Effects of emotional expression and fairness on LPP

In a repeated-measures ANOVA on the average amplitude in the LPP interval, with *fairness, electrode,* and *emotional expression* as factors, both *fairness, F*(2.51, 140.59) = 31.39, *MSE* = 24.39, *p* < .001, ${\boldsymbol{\eta}}_{\boldsymbol{p}}^{\mathbf{2}}$ = .36, and *emotional expression*, *F*(1.97, 110.36) = 16.58, *MSE* = 12.39, *p* < .001, ${\boldsymbol{\eta}}_{\boldsymbol{p}}^{\mathbf{2}}$ = .23, were significant. *Emotional expression* showed a smaller LPP after neutral (0.17 μV) than angry (1.08 μV) and happy (1.14 μV) expressions did. Fairness showed a negativity for fair conditions (−0.75 μV) as opposed to unfair (0.83 μV), offer size (1.22 μV), and very unfair (1.88 μV) ones. *Fairness* also interacted with *electrode, F*(3.40, 190.65) = 3.97, *MSE* = 3.59, *p* = .006, ${\boldsymbol{\eta}}_{\boldsymbol{p}}^{\mathbf{2}}$ = .07, showing the negativity associated with fairness as being most prevalent over the frontal site (−1.27 μV), whereas the positivity associated with the very unfair condition was most marked over the parietal site (2.66 μV).

Substituting *offer size* for *fairness* did not remove the effect of *offer size*, *F*(1.97, 110.10) = 9.71, *MSE* = 15.17, *p* < .001, ${\eta}_p^2$ = .15, nor the interaction between *offer size* and *electrode*, *F*(2.55, 142.75) = 6.47, *MSE* = 2.63, *p* = .001, ${\eta}_p^2$ = .10. (see Fig. 6)

### Effects of touch on offer perception

**Fig. 7:**
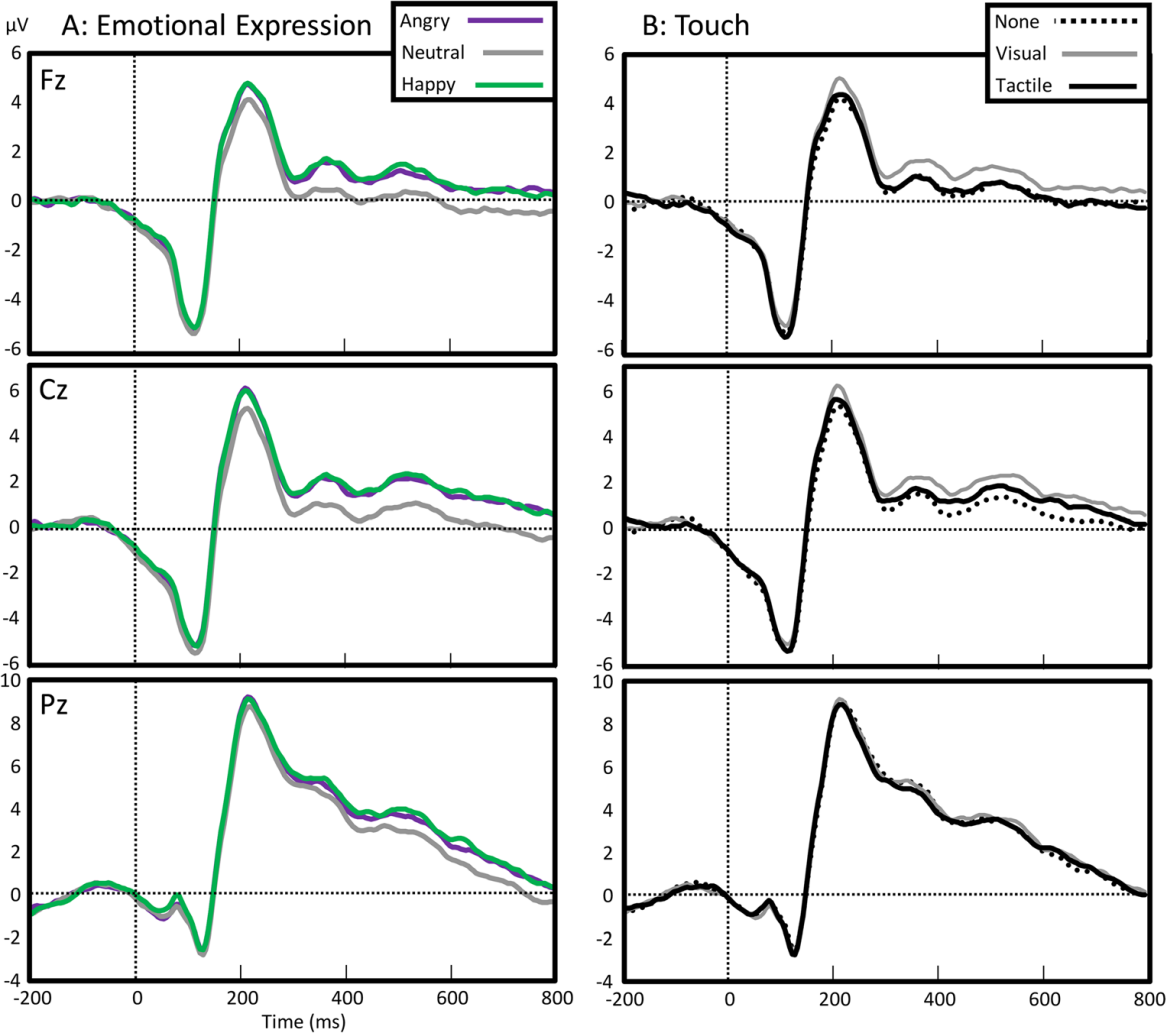
Event-related potential to offers as a function of nonverbal communication. Potentials are related to the emotional expression (left) and type of touch (right) displayed by the agent (left), but time-locked to and averaged across the four types of offers. (Colour figure online)

A repeated-measures ANOVA, with *time* (N1, MFN, P3, LPP), *electrode* (Fz, Cz, Pz), *fairness*, and *touch* as factors showed a significant effect of *touch*, *F*(1.88, 105.46) = 4.21, *MSE* = 34.34, *p* = .02, ${\eta}_p^2$ = .07. Visual touch conditions overall evoked higher amplitudes (0.92 μV) than no touch (0.49 μV) and tactile touch conditions (0.58 μV). *Touch* furthermore interacted with *electrode*, *F*(3.05, 170.66) = 10.57, *MSE* = 3.80, *p* < .001, ${\eta}_p^2$ = .16, generally showing stronger effects in frontal and central electrodes than parietal ones (see Fig. 7). A significant interaction was also observed between *touch* and *time*, *F*(4.24, 237.18) = 2.80, *MSE* = 5.12, *p* = .02, ${\eta}_p^2$ = .05, which in turn was modulated by *electrode*, *F*(5.03, 281.71) = 2.51, *MSE* = 0.96, *p* = .03, ${\eta}_p^2$ = .04.

To further inspect the effect of touch, we used separate three-way ANOVAs for each of the four potentials. Note that we omit from the report all redundant effects that have already been reported in the section titled Effects of Emotional Expression and Fairness on the ERP (e.g. the main effect of fairness). Please see the section titled Effects of Emotional Expression and Fairness on N1 for main effects and interactions between *time, electrode*, and *fairness.*

#### Effects of touch on N1

A repeated-measures ANOVA on the average amplitude for N1, with *electrode*, *fairness*, and *touch* as factors, showed no significant main effect of *touch*, *p* = .39, ${\boldsymbol{\eta}}_{\boldsymbol{p}}^{\mathbf{2}}$ = .02, power = .09. However, *touch* significantly interacted with *electrode*, *F*(2.79, 156.34) = 8.45, *MSE* = 0.94, *p* < .001, ${\boldsymbol{\eta}}_{\boldsymbol{p}}^{\mathbf{2}}$ = .13. The strongest effect of touch was observed over the frontal site, with more negativity after tactile touch (−4.74 μV) than after no (−4.58 μV) or visual (−4.32 μV) touch. *Touch* did not enter into any other interaction, *p*s > .25. As the N1 is generally more pronounced over frontal sites, this may indicate an enhanced N1 after tactile touch.

#### Effects of touch on MFN

A repeated-measures ANOVA on the MFN showed similar effects, with *touch* significantly interacting with *electrode*, *F*(2.99, 167.34) = 10.83, *MSE* = 1.17, *p* < .001, ${\boldsymbol{\eta}}_{\boldsymbol{p}}^{\mathbf{2}}$ = .16, but not as a main effect, *p* = .04, ${\boldsymbol{\eta}}_{\boldsymbol{p}}^{\mathbf{2}}$ = .06, power = .40. However, here, no touch and tactile touch showed negativity (1.72 μV, 1.79 μV, respectively) relative to visual touch (2.31 μV) over the frontal electrode, as well as over the central site (2.01 μV, 2.24 μV vs 2.62 μV). As shown in Fig. 4, few effects of touch were obtained over the parietal site. *Touch* did not interact with any other factor, *p*s > .12.

#### Effects of touch on P3

Similar ANOVAs on the P3 showed a significant main effect of *touch*, *F*(1.86, 103.92) = 5.66, *MSE* = 14.34, *p* = .006, ${\boldsymbol{\eta}}_{\boldsymbol{p}}^{\mathbf{2}}$ = .09, as well as an interaction between *touch* and *electrode*, *F*(2.99, 167.16) = 8.76, *p* < .001, ${\boldsymbol{\eta}}_{\boldsymbol{p}}^{\mathbf{2}}$ = .14. In general, visual touch evoked an amplified P3 (2.28 μV) relative to the tactile (1.82 μV) or no (1.64 μV) touch conditions. This effect was stronger in central (1.95 μV vs 1.44 μV and 0.96 μV) and frontal (1.33 μV vs 0.67 μV and 0.60 μV) sites, and virtually absent over the parietal site. *Touch* did not interact with any other factor, *p*s > .19.

#### Effects of touch on LPP

A final repeated-measures ANOVA on the LPP showed no significant main effect of touch, *p* = .03. Similar to the previous analyses, however, *touch* did interact with *electrode*, *F*(3.04, 170.09) = 5.93, *MSE* = 1.84, *p* = .001, ${\boldsymbol{\eta}}_{\boldsymbol{p}}^{\mathbf{2}}$ = .10. A pattern that was very similar to the P3 effects was observed: Visual touch evoked slightly more activity over frontal LPPs (0.66 μV) and central (1.16 μV) sites than no touch (0.10 μV, 0.30 μV) and tactile touch (0.10 μV, 0.81 μV) did. *Touch* did not interact with any other factor, *p*s > .53.

## Discussion

The aim of the present study was to investigate whether and when a messenger’s nonverbal behaviour influences our perception of what a message signifies. To this end, we presented artificial agents in virtual reality who displayed two distinct nonverbal communication channels—emotional expressions and interpersonal touch—prior to making economic proposals. In line with previous behavioural ultimatum game studies (Mussel et al., 2013), participants were found to reject unfair offers more than fair ones, and angry expressions increased this difference. Interpersonal touch was not found to promote compliance, contrary to studies reporting a Midas Touch effect (Guéguen & De Gail, 2003).

### Fairness perception and the MFN

The ERP analysis showed unfair offers evoked medial frontal negativity (MFN) relative to fair offers, which has previously been related to perception of a social norm of fairness being violated (Boksem & De Cremer, 2010). The present study provides evidence against a social-emotional interpretation of the MFN on two accounts. First, we show that although emotional expressions did affect the behavioural consequence to unfairness, they had no modulatory effect on the MFN. Second, we unexpectedly observed pronounced MFN to generous offers as much as unfair ones. In our discussion, we will first devote some attention to the implication of our results for fairness perception. We then discuss the degree to which nonverbal communication affects offer perception, as measured using other components of the ERP. Finally, we give an account of how the findings favour a late message–messenger integration account of neurosemiotics.

There are at least two explanations for the dissociations between behavioural responses and neural correlates, although neither fits well with the fairness norm violation account. A first possibility is that there are two stages of unfairness perception: a primary error response and a secondary *attribution* response. In this *error–attribution* model, a first process examines whether an economic offer is unfair or not without consideration for the target of the treatment—a generous offer is therefore still unfair. Only at a later stage do we disentangle whether this matters to ourselves, or whether it affects our interaction partner. This explanation is supported by the findings of De Bruijn and Von Rhein (2012), who showed that another’s mistakes provoke ERNs in the observer even if such mistakes hold positive significance for the observer (i.e., if the other’s mistake is made in a competitive context). A second, stronger explanation is that the MFN does not concern a detection of negative, erroneous outcomes, but rather qualifies a stimulus as not matching a receiver’s expectations regarding the message. Support for this expectancy-deviation account comes from studies examining the function of FRN in performance monitoring paradigms. These studies show that both unexpectedly positive and negative feedback regarding one’s performance amplify the FRN (e.g., Oliveira et al., 2007). Likewise, the FRN has also been observed in a shopping scenario in which valuable items were shown to have unexpectedly low prices (Schaefer, Buratto, Goto, & Brotherhood, 2016). In other words, the more parsimonious explanation suggests that the MFN in the ultimatum game is a correlate to a general “cold” classification of proposals as equal or not equal to a predicted stimulus.

### Nonverbal communication and offer perception

While neither nonverbal modality affected the MFN, we did observe emotional expressions to modulate later components related to offer perception. Following the MFN, during which unfairness itself was processed yet the type of unfairness was not, more fine-grained emotional appraisal of the offer took place after ca. 400 ms. Thus, at the stage of the P3, which has previously been shown to dissociate the probabilities of outcomes (Hajcak, Holroyd, Moser, & Simons, 2005) and prediction errors in decision-making tasks (e.g., Bellebaum & Daum, 2008), very unfair offers were dissociated from other proposals. Subsequently, at the time of the LPP, all four types of offers were finally dissociated, although at no point did they “economically” align: *Very unfair* and *generous* offers had an amplified LPP effect relative to the *unfair* and *fair* ones. This is theoretically interesting from the point of view that various studies used the LPP as a simple index of motivation (Bamford et al., 2015; Gable & Harmon-Jones, 2010). However, accounts suggesting a more evaluative role of the LPP (e.g., Schupp, Flaisch, Stockburger, & Junghöfer, 2006) were supported in our study, as both positive (generous) and negative (very unfair) conditions had relatively amplified LPPs.

The other nonverbal modality, touch, did not play a systematic role in offer processing or decision-making behaviour. Interpersonal touch does not, however, reliably affect decision-making. In our earlier studies, we found the physiological influences of symbolic touch to proposer feedback in the ultimatum game to occur later than expected (Spapé et al., 2015), while others found that even C-tactile optimized touch does not necessarily increase prosocial behaviour (Rosenberger, Ree, Eisenegger, & Sailer, 2018). Recently, we found the persuasive effect of touch to be strongly dependent on various situational and personality level factors (Harjunen, Spapé, Ahmed, Jacucci, & Ravaja, 2018). It seems therefore likely that the Midas Touch effect does not rely on an unconscious, automatic tendency to evaluate stimuli as being more positive, but is instead based on a complex appraisal process, which takes circumstances of the message and the messenger into account.

### Of the message and the messenger

The temporal dynamics of offer perception holds critical clues in the understanding of how the wider meaning of a message, in the context provided by the messenger, is decoded. Emotional expressions do not seem to modulate fairness perception in earlier (N1/MFN) processing stages. It therefore seems likely that the nature of the offer is decoded before the emotional expression is taken into account. As social expectations are critical for our development (Nummenmaa & Calder, 2009; Todd, Lewis, Meusel, & Zelazo, 2008), one might expect them to affect us very early on in terms of cognitive processing, such that an emotional expression causes top-down changes in expectations: A happy expression should naturally lead to a social norm of fairness or generosity. While changes in offer perception were indeed observed, they did not yet occur within the time range of the MFN (but see Ma, Qian, Hu, & Wang, 2017). Therefore, the present study suggests that contextual influences, such as facial emotional cues, manifest only at a relatively late stage, before which the offer is processed relatively independently of its context.

In conclusion, at an early stage of processing, fairness perception is paradoxically not very subjective. Of course, fairness is not an objective quality: Without a subject finding a message to not match their expectation, there would be no fairness. The study shows, however, that despite nonverbal communications changing how we respond to subsequent messages, our earliest fairness perception to these messages remains unaffected. Indeed, at this stage, evaluation appears to be a cold, rational process: We do not yet determine who the target of the unfairness is, and do not dissociate unfairness from generosity. Only after a message is decoded as dichotomously equal or unequal does the degree of subjective fairness become apparent, and only then does the nonverbal context become involved. Accordingly, nonverbal communication does not seem to immediately, inevitably affect the semiotics of the message and the messenger. In other words, we understand the meaning of “who says what” in precisely the other order: of the message, then the messenger.

## Electronic supplementary material

ESM 1 (DOCX 20 kb)

Supplementary Fig. 9 (PNG 1.77 MB)

High resolution image file (TIFF 7.93 MB)

## Footnotes

1 We mention them as MFN/FRN here, as in our view, both are similarly manifestations of the class of MFNs that arise from the perception that an outcome fails expectation (Gehring & Willoughby, 2004). Classically, FRN requires this expectation to be about one’s own performance (commonly a binary, positive or negative cue), while the MFN may be an interpersonal message. Thus, Boksem and De Cremer (2010) speak of MFN: Offers in their version of the ultimatum game do not concern a previous action from the participant, as each trial is seen as a single-shot decision game. Spapé, Hoggan, Jacucci, and Ravaja (2015), however, investigated how participants were affected by a computer’s decision to reject/accept their offer, accordingly focussing on the FRN, not the MFN. Although the subtle distinction is critical, it is not used consistently in the literature.

2 Of course, while running the ERP analysis over the full factorial five-way analysis is not optimal for the aforementioned reasons, it is certainly possible. The interested reader can verify the outcome of such an analysis in Supplementary Material 1.
