# Supplementary material for: The semiotics of the message and the messenger: How nonverbal communication affects fairness perception
Source: Cogn Affect Behav Neurosci. 2019 Jul 9;19(5):1259–72. doi: 10.3758/s13415-019-00738-8 (PMC6785596; doi:10.3758/s13415-019-00738-8)
Supplement: Supplementary file 1 — (DOCX 20 kb) [file 13415_2019_738_MOESM1_ESM.docx]

# 9.1 Supplemental material 1: Outcome of full factorial analysis

A repeated measures ANOVA on the average amplitude with *time* (N1, MFN, P3, LPP), *electrode* (Fz, Cz, Pz), *emotional expression* (angry, neutral, happy), *touch* (no touch, visual touch, visuo-tactile touch), and *fairness* (very unfair, unfair, fair, generous) showed significant main effects of *time,* F (2.42, 135.46) = 103.31, MSE = 541.64, p < .001, $\eta_{p}^{2}$ = .65, *emotional expression*, F (2, 112) = 14.16, p < .001, MSE = 93.09, $\eta_{p}^{2}$= .20, *touch*, F (2, 112) = 4.39, p = .01, $\eta_{p}^{2}$ = .07, but not offer size, p = .09. Significant interactions were observed between *time* and *electrode*, F (6, 336) = 28.24, p < .001, MSE = 50.91, $\eta_{p}^{2}$ = .34; between *time* and *offer size*, F (5.19, 290.73) = 33.94, p < .001, MSE = 24.85, $\eta_{p}^{2}$ = .38; between *electrode* and *offer size*, F (3.93, 219.88) = 4.17, p = .003, MSE = 9.11, $\eta_{p}^{2}$ = .07. These identified, successively, temporal and topographical differences between ERPs, and an effect of offer size that was qualified by time and electrode (see figure 2). *Electrode*, *time*, and *offer size* also entered into a significant three-way interaction, F (6.28, 351.39) = 11.07, p < .001, MSE = 2.42, $\eta_{p}^{2}$ = .16. With regards to *emotional expression* and *touch*, significant interactions were observed between *time* and *emotional expression*, F (4.69, 262.46) = 6.12, p < .001, MSE = 10.04, $\eta_{p}^{2}$ = .10, and between *electrode* and *emotional expression*, F (2.78, 155.79) = 3.62, p = .02, MSE = 7.61, $\eta_{p}^{2}$ = .06. These effects are described in more detail in section 3.2 and figure 4 A. Significant interactions were also observed between *time* and *touch*, F (4.24, 237.37) = 2.93, p = .02, MSE = 11.32, $\eta_{p}^{2}$ = .05; between *electrode* and *touch*, F (3.06, 171.24) = 10.60, p < .001, MSE = 8.71, $\eta_{p}^{2}$ = .16; and in the three-way interaction between *time*, *electrode,* and *touch*, F (4.95, 277.14) = 2.50, p = .03, MSE = 1.24, $\eta_{p}^{2}$ = .04.

Of particular interest to the present study were interactions between the non-verbal interaction channels – *emotional expression* and *touch* – on the one hand and *offer size* on the other. This was observed for *emotional expression,* with a significant 3-way interaction between *time, emotional expression*, and *offer size*, F (10.80, 604.58) = 2.74, p = .002, MSE = 8.19, $\eta_{p}^{2}$ = .05. *Touch*, however, did not enter in any further interaction with *offer size*, *p*s > .38, or *emotion*, *p*s > .18, or both, *p*s > .17. In section 3.2, we further investigated the effects of *emotional expression* by averaging epochs across *touch* conditions, maximising power and precision for detecting the particular interval at which this interaction occurs (section 3.2). For completeness, we also include the same analysis for the effect of *touch* (averaging epochs across *emotion* condition).

| Table 1 |
| --- |
| *Full factorial model results* |

| *Description* | *Factor* | *F* | *p* |
| --- | --- | --- | --- |
|  | ***T****ime* | 103.31 | **** |
| ***Main effects*** | ***E****lectrode* | 92.81 | **** |
|  | ***Offer*** *size* | 2.21 |  |
|  | ***Emo****tional expression* | 14.16 | **** |
|  | ***Touch*** | 4.39 | * |
|  | *T * E* | 28.24 | **** |
| ***Effects of fairness on the ERP x time and electrode*** | *T * Offer* | 33.94 | **** |
|  | *E * Offer* | 4.17 | *** |
|  | *T * E * Offer* | 11.07 | **** |
| ***Effects of emotion on the ERP*** | *T * Emo* | 6.11 | **** |
|  | *E * Emo* | 3.62 | * |
|  | *T * E * Emo* | 1.75 |  |
| ***Effects of emotion on fairness perception*** | *Offer * Emo* | 2.05 |  |
|  | *T * Offer * Emo* | 2.74 | *** |
|  | *E * Offer * Emo* | 1.39 |  |
|  | *T * E * Offer * Emo* | 0.93 |  |
| ***Effects of touch on the ERP*** | *T * Touch* | 2.93 | * |
|  | *E * Touch* | 10.60 | **** |
|  | *T * E * Touch* | 2.50 | * |
| ***Effects of touch on fairness perception*** | *Offer * Touch* | 0.90 |  |
|  | *T * Offer * Touch* | 1.07 |  |
|  | *E * Offer * Touch* | 0.93 |  |
|  | *T * E * Offer * Touch* | 0.99 |  |
|  | *Emo * Touch* | 0.82 |  |
| ***Other interactions*** | *T * Emo * Touch* | 0.74 |  |
|  | *E * Emo * Touch* | 1.54 |  |
|  | *T * E * Emo * Touch* | 1.38 |  |
|  | *Offer * Emo * Touch* | 0.93 |  |
|  | *T * Offer * Emo * Touch* | 0.97 |  |
|  | *E * Offer * Emo * Touch* | 0.36 |  |
|  | *T * E * Offer * Emo * Touch* | 0.76 |  |

| *Note.* Results of the full-factorial, five-way repeated measures ANOVA. Explanations of abbreviations of factors are underlined. The notes provide brief explanations of the tests within the present framework. Their significance was tested with Greenhouse-Geisser adjustments where appropriate, with *: p < .05; **: p < .01; ***: p < .005, ****: p < .001. |
| --- |
|  |
|  |
|  |

# 9.2 Supplemental material 2: Emotional expressions and fairness, a complete analysis.

# The presented figure shows the outcome of separate repeated measures ANOVAs with *fairness* (very unfair, unfair, fair, generous) and *emotional expression* (anger, neutral, happy) as factors. These were run on a total of 679 measures, all amplitude averages over 10 ms bins, from 180 before to 780 ms, for 7 electrodes (Fz, FC1, FC2, Cz, CP1, CP2, and Pz). Colours on top of each graph in the middle column indicate significance of the main effect of *emotional expression* (in black), of the main effect of *fairness* (in red), and of the interaction between *fairness* and *emotional expression* (in blue), the shade indicating the level of significance, with a threshold at p < .025. Note that these tests are deliberately not corrected for multiple comparisons so as to indicate the most liberal interpretation of the data. In other words, the presented graphs may show many false positives – the reliability of very early effects is therefore very low. However, note that the first point at which the interaction between fairness and emotional expression reaches significance, is relatively late, even with these liberal criteria. The presented evidence thus shows 1) that emotional modulations of the fairness effect occurs *after the fairness effect itself*, and 2) that such modulations seem to principally occur over frontal sites.
