# Supplementary figures and images for: The semiotics of the message and the messenger: How nonverbal communication affects fairness perception
Source: Cogn Affect Behav Neurosci. 2019 Jul 9;19(5):1259–72. doi: 10.3758/s13415-019-00738-8 (PMC6785596; doi:10.3758/s13415-019-00738-8)

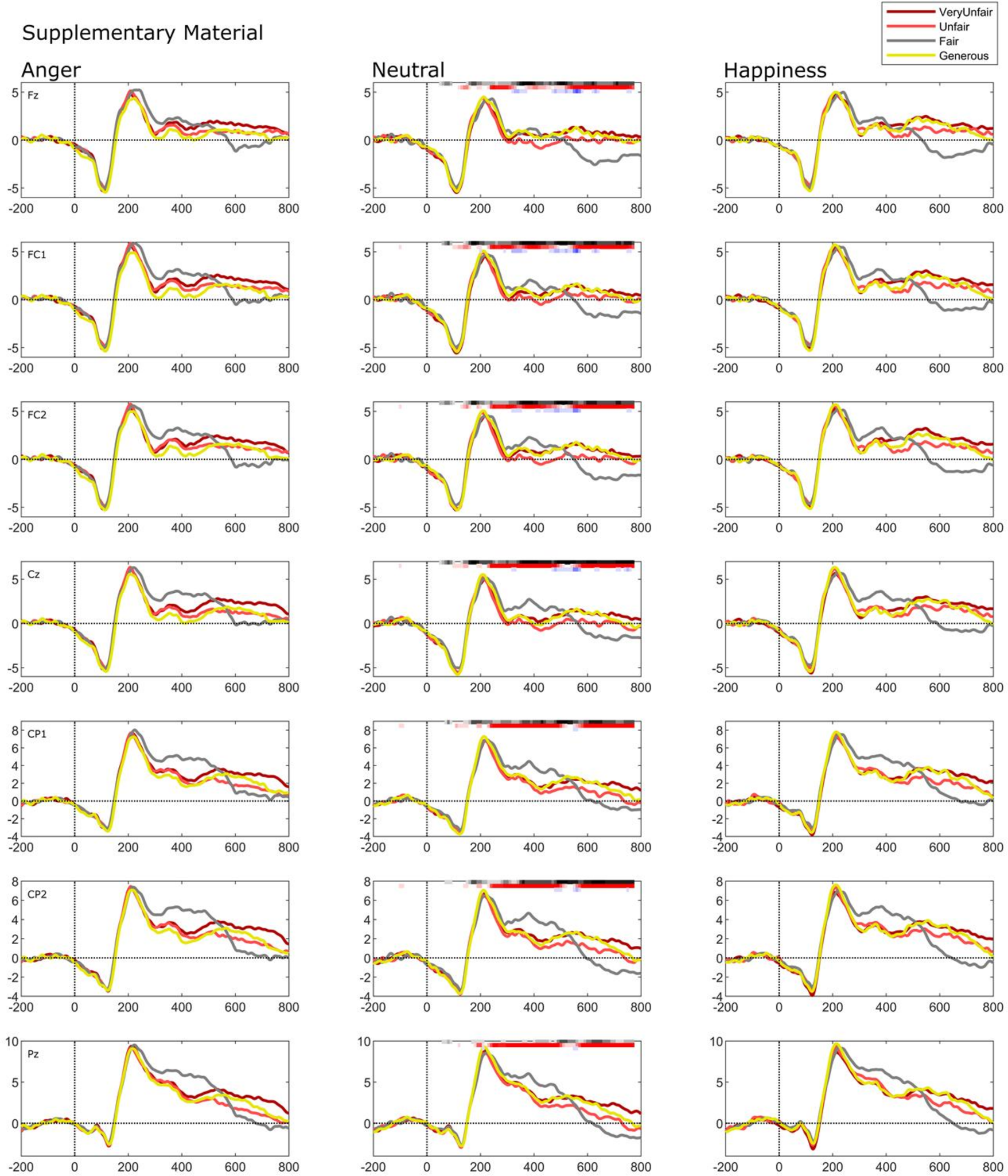

Supplement: Supplementary file 2 — (PNG 1.77 MB) [file 13415_2019_738_Fig9_ESM.png]

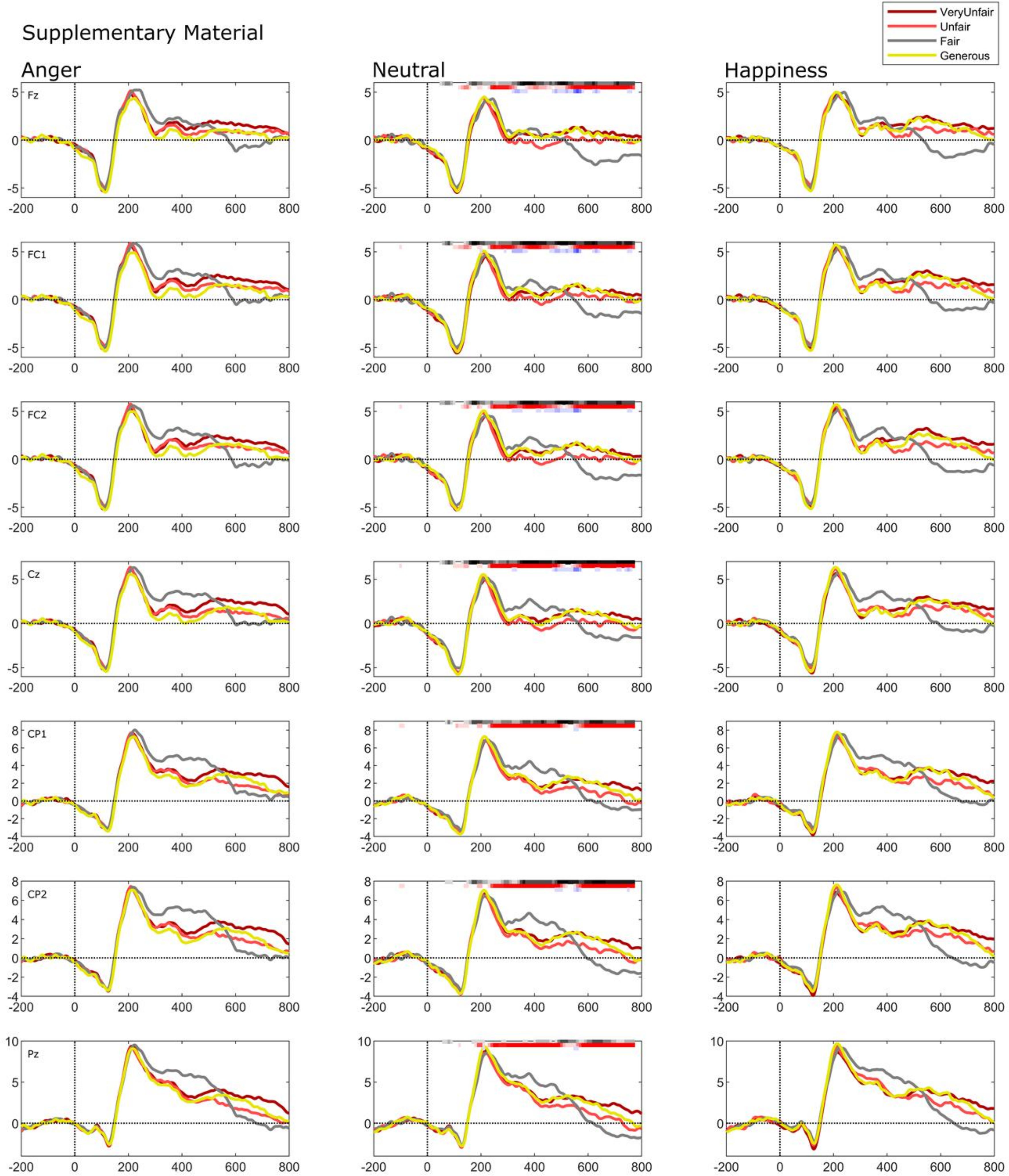

Supplement: Supplementary file 3 — High resolution image file (TIFF 7.93 MB) [file 13415_2019_738_Fig9_ESM.tiff]
